# Current Issues and Future Perspectives of Targeted Therapies in Primary Mediastinal Large B-Cell Lymphoma

**DOI:** 10.3390/jcm14041191

**Published:** 2025-02-11

**Authors:** Athanasios Liaskas, Maria N. Dimopoulou, Alexia Piperidou, Maria K. Angelopoulou, Theodoros P. Vassilakopoulos

**Affiliations:** Department of Hematology and Bone Marrow Transplantation, Medical School, National and Kapodistrian University of Athens, General Hospital of Athens “Laikon”, 11527 Athens, Greece; ath.liaskas@gmail.com (A.L.); alexia_piper@hotmail.gr (A.P.); mkangelop@gmail.com (M.K.A.); theopvass@hotmail.com (T.P.V.)

**Keywords:** primary mediastinal large B-cell lymphoma, brentuximab vedotin, nivolumab, pembrolizumab, CAR T-cells

## Abstract

Primary mediastinal large B-cell lymphoma (PMLBCL) is a rare, aggressive B-cell lymphoma, sharing common features with diffuse large B-cell lymphoma (DLBCL) and Hodgkin lymphoma (HL). PMLBCL is usually cured with single-hit immunochemotherapy in the first-line setting. Relapses tend to be aggressive and may be unresponsive to conventional chemotherapy. Autologous stem cell transplant (ASCT) remains a viable option for chemosensitive patients; nevertheless, targeted therapies appear to be highly promising. Checkpoint inhibitors (CPIs) have already transformed the course of relapse/refractory disease, while CD-19-directed Chimeric Antigen Receptor (CAR) T-cell therapy may produce remarkably favorable outcomes. The exact position of CAR T-cells and CPIs in the treatment algorithm, along with the role of radiotherapy and ASCT, remains to be precisely determined. In the current review, we aim to present the recent research on targeted agents in PMLBCL and define their sequencing within the treatment algorithm, mainly in the relapse/refractory setting.

## 1. Introduction

Primary mediastinal large B-cell lymphoma (PMLBCL) is a rare, aggressive B-cell lymphoma originating from thymic B-cells. It accounts for 2.4% of all non-Hodgkin lymphomas, affecting predominantly young females with a median age of 35 years at presentation [1]. PMLBCL shares common biological features with diffuse large B-cell lymphoma (DLBCL) and nodular sclerosing Hodgkin lymphoma (HL). Tumor cells express B-cell markers (CD20, CD19), which currently serve as therapeutic targets, and frequently CD30, which can also be targeted by the immuno-conjugate brentuximab vedotin (BV). According to gene expression profiling studies, the molecular and transcriptional signature of PMLBCL is closer to that of HL and involves two main pathobiological milestones: the abnormal activation of the NF-κB and JAK/STAT signaling pathways and the development of an immune-anergic microenvironment [2,3]. The cytogenetic basis of these deregulations is the presence of copy number amplifications and translocations at the 9p24.1 locus, which contains both JAK2 and the Programmed Death Ligands 1 and 2 (PDL-1, PDL-2). These aberrations lead to overexpression of PDL-1 and PDL-2 and, subsequently, to T-cell exhaustion and immune evasion [2,3]. Based on this, PD-1 blockade is currently a main therapeutic option for relapsed/refractory (R/R) disease, as discussed below.

Patients usually present with bulky mediastinal lymphadenopathy, often causing various oncologic emergencies, such as superior vena cava syndrome, pericardial or pleural effusion, and airway compression [4]. Advanced-stage disease with the involvement of peculiar extranodal infradiaphragmatic sites at diagnosis is rare [5]. Both such lesions and Central Nervous System (CNS) localizations are more commonly seen in the R/R setting [6,7].

### 1.1. Current Practices and Controversies in First-Line Therapy

The optimal first-line treatment is not precisely established, as there have been no randomized trials comparing the various available regimens, and only a single non-inferiority trial has explored the need for consolidative radiotherapy (RT) [8]. Prior to the rituximab era, the CHOP regimen (cyclophosphamide, doxorubicin, vincristine, prednisone) plus RT was the standard of care, with clearly suboptimal results [9,10,11]. Despite other intensive regimens being apparently more effective, they have never been compared in the context of randomized trials [12,13,14,15]. The addition of rituximab to the conventional CHOP regimen—mostly followed by RT—led to a significant increase in long-term disease control, exceeding 75%, compared to ~50% achieved by CHOP + RT [16,17,18]. A dose-dense approach with R-CHOP delivered every 14 days (R-CHOP-14) was associated with better efficacy than conventional R-CHOP-21 in some studies, but the use of consolidative RT and other therapies was heterogenous. Additionally, the finding was not confirmed in other studies, including the UNFOLDER randomized trial for lower-risk patients [19,20,21,22]. The National Cancer Institute (NCI) developed an intensive dose-adjusted regimen with etoposide, doxorubicin, cyclophosphamide, vincristine, prednisone, and rituximab (R-DA-EPOCH) without RT. In a phase II trial of 51 patients, R-DA-EPOCH exhibited outstanding efficacy, with event-free survival exceeding 90% [23]. Although real-life studies may be less enthusiastic, the results of R-DA-EPOCH overall appear better or marginally better than those of R-CHOP-21 [24,25,26,27,28]. Importantly, R-DA-EPOCH exempts patients from mediastinal RT, which could be reserved for the small minority of patients with inadequate metabolic response at the end of treatment (EoT) [29]. However, R-DA-EPOCH remains an intensive regimen with significant acute hematologic toxicity and possibly a small but not negligible risk of secondary myeloid neoplasms [30].

### 1.2. Treatment of Relapsed/Refractory Disease

R/R disease occurs early, typically within a few months of treatment completion, and tends to be aggressive, often with extranodal involvement [7,31]. The optimal treatment approach still remains undefined. Similar to the case for DLBCL patients, the standard and, until recently, only available option for R/R patients was salvage chemotherapy and subsequent autologous stem cell transplant (ASCT). Kuruvilla et al. reported high rates of salvage failure and poorer post-ASCT outcomes in patients with PMLBCL compared to DLBCL [32]. However, the strongest predictor of long-term remission is chemosensitivity prior to transplant, and several retrospective studies have shown that roughly two-thirds of the patients are chemosensitive, with 40% of patients with R/R disease achieving CR with salvage chemotherapy; cure rates with ASCT may be as high as 70% for chemosensitive patients but no more than 40–45% for those who proceed to ASCT despite chemorefractoriness [33,34,35,36,37]. Overall, ~60% of transplanted patients might be cured, but it should be underlined that a sizeable subgroup of chemorefractory patients could not undergo ASCT prior to the era of novel therapeutics and had very poor outcomes. In our PMLBCL series, the long-term survival rate for unselected patients with R/R disease (whether receiving ASCT or not) was 45%, with the vast majority of them treated prior to the era of novel therapies (unpublished data). In case of chemorefractoriness or relapse after ASCT, therapeutic options have been limited in the past, thus leading to very high disease-related mortality, as allogeneic stem cell transplant may be effective only in a highly selected minority of patients [38].

Recently, based on the distinct pathobiology of PMLBCL, including the expression of CD30, PDL-1, PDL-2, and B-cell markers, targeted therapies were tested and eventually became available in the R/R setting [39]. The aim of this review is to describe the recent advances in targeted therapeutics in PMLBCL, the treatment algorithm, and future perspectives.

## 2. Programmed Death-1 (PD-1) Blockade

### 2.1. Pembrolizumab

PD-1 receptors are located on the surface of normally activated T-cells. Antigen-presenting cells express PDL-1, which engages PD-1 receptors on T-cells, leading to attenuation of the T-cell receptor (TCR) signaling pathway and eventually impeding T-cell activation, proliferation, cytokine production, and cytotoxicity [40]. Tumor cells in PMLBCL overexpress PDL-1 due to amplifications and translocations at the 9p24.1 locus, forming an immune-privileged environment [41].

Under this rationale, PD-1 blockade was first evaluated in the phase Ib KEYNOTE-013 trial. Pembrolizumab, a PD-1 inhibitor, was given to 17 patients who were ineligible for or had relapsed after ASCT. The overall response rate (ORR) was 41%, with two patients achieving Complete Response (CR), which was rather impressive considering that this subgroup of patients had, until then, an extremely poor prognosis [42]. In an update, after a median follow-up of 29.1 months, the median duration of response (DOR) was not reached. The median progression-free survival (PFS) was 10.4 months, with a 1-year PFS rate of 47%. The median overall survival (OS) was 31.4 months, with a 1-year OS rate of 65% [43].

KEYNOTE-170 was a phase II study aiming to confirm these results. Pembrolizumab was delivered every 3 weeks to 53 patients with R/R PMLBCL (approximately one-third being primary refractory) for up to 35 cycles, corresponding to a 2-year treatment duration. Similar to that for KEYNOTE-013, the ORR was 43%, with 13% of patients achieving CR and 32% achieving Partial Response (PR), of which seven patients subsequently improved to CR. Grade 3–4 treatment-related adverse events occurred in 23% of patients, with no patients discontinuing treatment [44]. The final analysis of the trial highlighted the durable responses of pembrolizumab as, with a median follow-up time of ~4 years, the median DOR was not reached, and none of the patients with CR progressed. Overall, the 4-year PFS and OS rates were 33% and 45.3%, respectively [45]. The study confirmed that pembrolizumab is effective and can offer long-term disease control in responding patients. In KEYONTE-170, an increase in PDL-1 expression was associated with copy gains in the 9p24.1 locus, rather than other cytogenetic aberrations, and a high PDL-1 expression score (H-score) was associated with better outcomes, without reaching statistical significance [44].

Pembrolizumab was also evaluated in a small Japanese population of seven patients with R/R PMLBCL, with efficacy and safety results consistent with those of KEYNOTE-170 [46].

Based on data on solid tumors, Keynote-B68 aimed to explore the efficacy of delivering pembrolizumab at a dose of 400 mg every 6 weeks as a more convenient dose schedule. The study also included patients with R/R HL and enrolled only six patients with R/R PMLBCL. In the PMLBCL population, the ORR was 50%, and the median DOR was 9.7 months at a median follow-up time of 17.7 months. The 1-year PFS rate was 33.3%. More patients and follow-up are needed in order to evaluate the anti-tumor activity of this treatment schedule [47].

### 2.2. The Combination of Brentuximab Vedotin and Nivolumab (BV + nivo)

BV is an anti-CD30 monoclonal antibody conjugated to monomethyl auristatin E, a cytotoxic anti-tubulin agent. BV promotes apoptosis in CD30+ tumor cells and has exhibited remarkable efficacy in HL [48,49,50,51,52,53,54]. Based on the variable expression of CD30 in PMLBCL, BV was administered as a monotherapy in a small trial of 15 patients with R/R disease, but the response rates were disappointing, with only 2/15 (13.3%) patients achieving PR and none achieving CR [55]. Despite the disappointing result, the failure of BV monotherapy does not preclude its use in PMLCL.

In fact, there is preclinical evidence that BV promotes the depletion of intratumoral immunosuppressive CD30+ regulatory T-cells and the expansion of CD8+ T-cells, resulting in immunogenic cell death in tumor cells and possibly enhancing the effects of PD-1 blockade [56,57]. This was the rationale of Checkmate 436, a phase I/II study aiming to evaluate BV + nivo in patients with R/R PMLBCL. BV was given at a dose of 1.8 mg/kg (maximum 180 mg) in combination with a 240mg flat dose of nivolumab every 21 days until progression. Among the 30 patients enrolled in the study, the majority (67%) were ineligible for ASCT due to chemorefractory disease. The ORR was 73% (*n* = 22), with 37% of patients achieving CR (*n* = 12) [58]. At a median follow-up of 39.6 months, the median DOR was not reached, the 2-year PFS rate was 55.5%, and the 2-year OS rate was 75.5%. Out of 22 responding patients, 4 remained disease-free without any further treatment, while 18 patients received subsequent treatment, of which 7 received consolidative RT. Overall, 12 patients underwent consolidative autologous or allogeneic transplant; remarkably, the 100-day CR rate for either group was 100% [59].

BV + nivo was given to 10 patients with R/R PMLBCL (2 of whom had participated in Checkmate 436) in a small single-center study. The patients’ median age was 34, 73% were males, 50% had extranodal disease at diagnosis, and 80% were primary refractory. One patient had undergone ASCT. The best ORR was 70%, with 40% achieving CR after four cycles. The 3-year PFS and OS rates were 64% and 70% respectively. None of the patients proceeded to ASCT. Interestingly, five patients with localized relapse received consolidative RT, and all remained disease-free at 3 years [60]. These findings, along with the results of Checkmate 435, could possibly shape a new treatment algorithm with curative intent; BV + nivo could serve as a salvage combination prior to ASCT, bridging to Chimeric Antigen Receptor (CAR) T-cells or even to RT for patients with localized disease. However, this small series suggests that BV + nivo ± RT could also be a “final” curative treatment. This should be evaluated in further studies.

The summarized results of the three largest studies, KEYNOTE-013, KEYNOTE-170, and Checkmate 436, are presented comparatively in Table 1. It is unknown which of these treatments is optimal and in which setting. BV + nivo produced better response rates in Checkmate 436. However, the patient characteristics were different among the three studies; patients in KEYNOTE-170 were younger and more heavily pretreated but were less frequently primary refractory. More experience is needed to draw conclusions about the comparative long-term disease control rates.

Based on the above very promising results in the R/R setting, BV + nivo will be evaluated in the frontline setting, in combination with R-CHOP, within the PACIFIC trial, which is currently in progress [66]. On the contrary, although R-pembro-CHOP is feasible and safe in previously untreated DLBCL, there is no current study in PMLBCL [67,68].

### 2.3. CD19-Directed CAR T-Cell Therapy

Patients with PMLBCL were included in the two pivotal trials ZUMA-1 and TRANSCEND NHL 001, along with other large B-cell histologies [62,69] in patients who had R/R disease after two or more lines of treatment. However, their results refer to a study population mainly consisting of patients with DLBCL; therefore, it is unclear whether these findings are equally applicable to patients with PMLBCL. Overall, eight patients were included in the ZUMA-1 trial and received Axicabtagene Ciloleucel (axi-cel). The ORR was 83% with 71% CR for the combined group of patients with PMLBCL and transformed follicular lymphoma [69]. Within a median follow-up of 27 months, ongoing responses were maintained in five of the eight patients with PMLBCL [61], and the median event-free survival was still not estimable in the 5-year follow-up report [70]. In TRANSCEND NHL 001, 14 patients with R/R PMLBCL were enrolled and received Lisocabtagene Maraleucel (liso-cel). The ORR was 78.6% for patients with PMLBCL, while the median DOR, PFS, and OS were not reached [62]. The JULIET trial of the CAR T product tisagenlecleucel did not include patients with PMLBCL at all [71]. These findings led to the approval of axi-cel and liso-cel by the US FDA for the treatment of primary refractory or R/R PMLBCL after two lines of therapy.

It is now recommended that primary refractory or early relapsing (<12 months) patients with DLBCL should be treated with axi-cel or liso-cel, rather than ASCT, based on the results of the ZUMA-7 and TRANSFORM trials [72,73,74]. However, it is unknown whether this is the case for PMLBCL patients, especially if we take into account the efficacy of checkpoint inhibitors. In fact, ZUMA-7 did not include patients with PMLBCL, whereas 18 patients were enrolled in TRANSFORM (8 were treated with liso-cel) but with no subgroup analysis reported [73]. The BELINDA trial, in which tisa-cel failed to achieve better results than ASCT in patients with DLBCL, included 25 patients with R/R PMLBCL (12 of whom received tisa-cel), with no significant differences in survival compared to other histologies [75]. Briefly, large pivotal trials could not provide robust data about the efficacy of CAR T-cells due to the small number of patients. Even more important answers are to be given regarding the comparative efficacy among ASCT, CAR T-cells, and checkpoint inhibitors, as well as their optimal sequencing in the R/R setting.

Crombie JL et al. reported a large multicenter study including 33 patients with R/R PMLBCL who were treated with axi-cel outside the clinical trial setting. Most of the patients had received prior RT, and almost one-third had undergone ASCT. The results were encouraging as the ORR was 76% with a 67% CR rate in the intent-to-treat population. The 2-year PFS and OS rates were 64% and 78%, respectively. Interestingly, this study incorporated checkpoint inhibitors either prior to axi-cel infusion (14 patients), post-axi-cel infusion (4 patients), or both (1 patient). The ORR was better in patients who were treated with checkpoint inhibitors within 100 days following axi-cel compared to pre-CAR T-cell administration, with the ORR being 75% and the CR rate 100%, versus 40% and 27%, respectively. However, this sample was relatively small, and the results did not reach statistical significance [63].

A few real-life studies have aimed to compare the efficacy of CAR T-cells among large B-cell histologies. The efficacy of axi-cel in PMLBCL compared to other large B-cell lymphomas was evaluated within the CART-SIE real-life study in which 260 patients received axi-cel, including 70 patients with PMLBCL and 135 with DLBCL-NOS, transformed follicular lymphoma, and high-grade B-cell lymphoma. The patients with PMLBCL had significantly more frequently refractory and bulky disease, and almost equal numbers needed bridging therapy in both groups. The response rates at day +90 were significantly superior for PMLBCL patients compared to all other large B-cell histologies, with an ORR of 77% including a 53% CR rate, versus 54% and 47%, respectively, for other large-B-cell lymphomas. The median DOR was not reached in patients with PMLBCL. The 1-year PFS and OS rates were significantly better for PMLBCL patients, with reported rates of 62% vs. 48% and 86% vs. 71%, respectively. A survival benefit was also demonstrated in a multivariate analysis [64]. Under a similar rationale, a GLA/DRST registry study included 13 patients with R/R PMLBCL and 131 with R/R DLBCL who received axi-cel. Both groups had comparable patient characteristics. Overall, 92% of the PMLBCL patients had active disease prior to lymphodepletion, mostly due to unsuccessful bridging. Patients with PMLBCL had better ORRs (85 vs. 71%) and CR rates (54% vs. 42%) but without reaching statistical significance. Notably, the 2-year PFS rate was significantly superior for PMLBCL patients (54% vs. 26%) and reached a plateau beyond 8 months after the infusion. The PFS also remained satisfactory regardless of bridging [65]. These findings point towards better efficacy and, possibly, disease control in the long term, but the size of the reported population remained small. The summarized results of pivotal and real-life studies of CAR T-cells are presented in Table 1.

### 2.4. Other Novel Approaches

Novel monoclonal antibody–drug conjugates and bispecific antibodies have already been incorporated into the therapeutic algorithm for R/R DLBCL. Unfortunately, patients with R/R PMLBCL are usually excluded or underrepresented in pivotal studies. In the LOTIS-2 trial, the efficacy and safety of loncastuximab tesirine, an anti-CD19 antibody conjugated to a pyrrolobenzodiazepine dimer cytotoxin, was evaluated in 145 patients with R/R DLBCL, including only 7 patients with R/R PMLBCL. The ORR was 48.3% with a 24% CR rate in the whole patient population, without separate data for patients with PMLBCL [76]. Glofitamab and epcoritamab are both CD3xCD20 bispecific antibodies with remarkable efficacy in patients with R/R DLBCL., About 40% of patients achieved CR with both agents [77,78]. However, only 4% and 2.5% of the patient populations treated with glofitamab and epcoritamab, respectively, had PMLBCL, with no subgroup analysis in either study, thus precluding any conclusions regarding these promising bispecific antibodies in PMLBCL. None of the registrational studies of polatuzumab vedotin [79,80] included patients with PMLBCL, and the same is true for the ELM-1 trial of odronextamab [81], the L-MIND trial of tafasitamab–lenalidomide [82], and SADAL for selinexor [83].

### 2.5. The Current Treatment Algorithm

**Figure 1:**
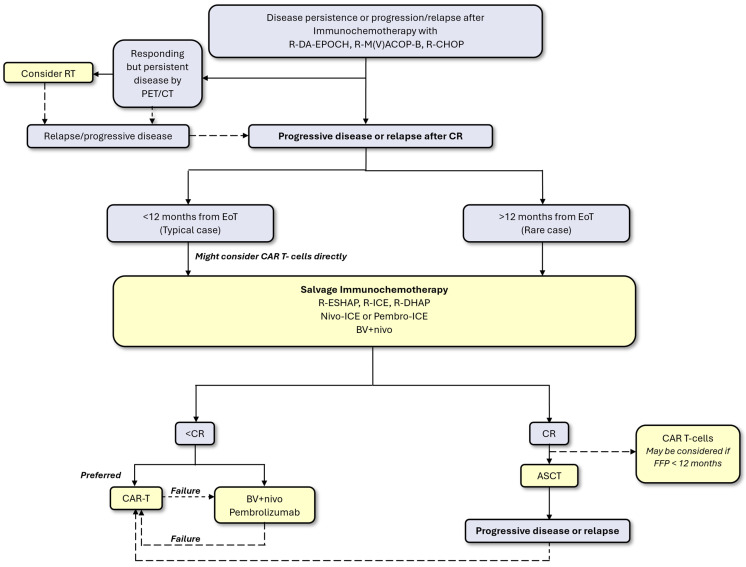
Proposed treatment algorithm for relapsed/refractory primary mediastinal large B-cell lymphoma.

Currently, there is no established algorithm for the optimal treatment of R/R PMLBCL. A proposed treatment algorithm is depicted in Figure 1.

For patients with a partial metabolic response and Deauville score of 5 (markedly increased uptake compared to the liver and/or new lesions) in the EoT-PET, i.e., those without clinical or conventional radiographic progression, salvage RT could be a curative option, especially after R-da-EPOCH but after R-CHOP as well [29,84]. Typically, such patients with non-progressive but persistent and responding disease have a Deauville score of 5 with a moderately high SUVmax of <10–15 [28].

In the absence of dedicated PMLBCL guidelines, patients with progressive or relapsing disease could be treated based on DLBCL strategies in real life [85,86], but this is not based on formal evidence. In the rare event of late relapse occurring >12 months from treatment completion, salvage chemotherapy and ASCT remain a potentially curative option if a response can be obtained before transplant. However, in PMLBCL, disease progression almost invariably occurs less than 12 months from EoT [17], and almost all patients are potentially eligible for ASCT and CAR T-cell treatment due to their young age. For this usual clinical scenario of primary refractory disease or early relapse—and based on the above considerations—almost all patients could be referred to CAR T-cell therapy. Liso-cel is officially approved for patients relapsing within 12 months of treatment completion but is not available in many countries yet; axi-cel is not formally approved for R/R PMBCL in the second line. However, exactly within this primary refractory or early relapsing population, chemosensitive patients are not infrequent [33,34,35,36] and can still be cured with ASCT with considerable success, as described above.

PD-1 inhibitors could be implemented in various ways prior to ASCT or CAR T-cell therapy. Pretransplant salvage regimens incorporating nivolumab or pembrolizumab have been successfully developed in HL and could be a reasonable approach as a pretransplant salvage therapy in PMLBCL [87,88,89,90,91], instead of their classical rituximab-containing counterparts [92,93,94]. The incorporation of rituximab in such PD-1-inhibitor-containing regimens has not been studied. The BV + nivo chemo-free approach could also be used as a bridging-therapy in order to achieve CR before a potentially curative treatment such as ASCT or even CAR T-cells [59,60]. Another interesting approach is offering PD-1 inhibitors to patients who relapse after CAR T-cells. PD-1 blockade leads to a reversal of CAR T-cell exhaustion and increases CAR T-cell activation and proliferation [95,96]. There are reports of successful long-term disease control with pembrolizumab in patients relapsing after CAR T-cell treatment [97,98]. In a large cohort of 96 patients with R/R aggressive B-cell lymphomas who received checkpoint inhibitors after CAR T-cell failure, the ORR was 19%, with 10% CR. Interestingly, the eight patients with R/R PMLBCL had a significantly better ORR of 63%, as well as superior PFS compared to other large B-cell histologies [99].

CNS relapses are quite infrequent, with an incidence rate of 2.3–3.8%; they tend to be isolated to the CNS [6,31]. The standard-of-care approach involves methotrexate-containing regimens, followed by ASCT with a CNS-penetrating conditioning regimen. However, this strategy is associated with poor disease control and compromised survival [100,101,102]. PD-1 blockade has been successful in patients with R/R primary CNS lymphoma [103]. In a case report, pembrolizumab was shown to produce an adequate response and served as a bridging therapy towards allo-SCT [104]. Nivolumab has also been used in single cases [105]. Finally, CAR T-cell therapy may also cure CNS disease in DLBCL, albeit with limited success, while there is no organized experience in PMLBCL [106,107].

### 2.6. Future Perspectives

Overall, the level of evidence in the aforementioned studies remains rather low due to the relatively small population size and the lack of randomized control trials. Thus, it is difficult to produce robust conclusions and recommendations. Several questions still remain unanswered despite the great progress made between 2001 and 2024 in PMLBCL. Improving the efficacy and reducing the toxicity of first-line therapy still remains a partially assessed issue. Dedicated data on the incorporation of PD-1 inhibitors in this setting might be helpful [67,108]. The long-term toxicity of chemotherapy and RT especially in PMLBCL is projected from data on Hodgkin lymphoma. Unfortunately, the literature on late effects in PMLBCL is very limited [30,109], so cooperative efforts with long follow-ups are needed. The incorporation of PD-1 inhibitors in the second line should also be specifically evaluated. A major issue will be the definition of the optimal sequencing of CAR T-cell therapy, ASCT, and PD-1 inhibitors. Finally, further elucidation of the complex genomic landscape of PMLBCL could improve prognostication and treatment decision-making in the near future. Sequencing studies and mutational analyses in pretreatment samples of a relatively large cohort of patients with PMLBCL revealed recurrent pathway lesions with clear prognostic and therapeutic implications [110]. The circulating tumor DNA load has been used alone or in combination with traditional clinical prognostic factors in order to define prognosis or treatment response [111,112,113,114,115,116].

## 3. Conclusions

PMLBCL is usually cured with single-hit immunochemotherapy in the first-line setting, sometimes followed by RT. The disease progression tends to be clinically aggressive and may be unresponsive to conventional chemotherapy. Although ASCT still remains a viable option for chemosensitive patients, targeted therapies appear to be highly promising. PD-1 inhibitors have already transformed the course of R/R disease. CD-19-directed CAR T-cell therapy may produce remarkably favorable outcomes. The exact position of CAR T-cells and PD-1 inhibitors in the treatment algorithm, along with the role of RT and ASCT, remains to be precisely determined. Patients with R/R PMLBCL appear to be homogenous in terms of timing or relapse and their potential eligibility for ASCT and CAR T-cells, facilitating the design of clinical trials in the future. However, even today, despite high but still imperfect disease control rates with immunochemotherapy, with or without RT, the implementation of novel targeted approaches has greatly minimized disease-related deaths in PMLBCL.

## Figures and Tables

**Table 1:** Summarized results of the main studies incorporating novel agents in R/R PMLBCL.

	KEYNOTE-013 [44]	KEYNOTE-170 [45]	CHECKMATE 436 [59]	ZUMA-1 [61]	TRANSCEND NHL 001 [62]	Crombie JL et al. [63]	CART-SIE [64]	GLA/DRST [65]
**Study Phase**	Ib	II	I/II	II	II	Real-life	Real-life	Real-life
**Regimen**	Pembrolizumab	Pembrolizumab	Brentuximab vedotin plus nivolumab	Axi-cel	Liso-cel	Axi-cel	Axi-cel	Axi-cel
**Patients, N**	17	53	30	30 * (8 with PMBCL)	15	33	70	13
**Median age (range)**	30 (22–62)	33 (20–61)	36 (19–83)	57 (23–76) *	63 (54–70) ^§^	32 (18–46)	33 (IQR:27–41.8)	39 (20–48)
**Males (%)**	5 (28%)	23 (43%)	13 (43.3%)	18 (75%) *	174 (65%) ^§^	NA	35 (51%)	7 (54%)
**Median number of prior lines (range)**	3 (2–6)	3 (2–8)	2 (2–5)	NA	3 (2–4) ^§^	3 (1–7)	2 (2–3)	3 (2–4)
**Primary refractory (%)**	8 (44%)	16 (30.2%)	18 (60%)	0	181 (67%) ^§^	NA	63 (90%)	12 (92%)
**Prior ASCT (%)**	6 (33%)	14 (26.4%)	4 (13.3%)	5 (21%) *	90 (33%) ^§^	10 (30%)	12 (17%)	0
**Prior RT (%)**	11 (61%)	17 (32.1%)	NA	NA	NA	22(67%)	NA	2 (18%)
**ORR, %**	41.2%	41.5%	73.3%	83% *	78.6%	78%	69%	85%
**CR, %**	11.8%	20.8%	40%	71% *	50%	69%	65%	54%
**Median follow-up, months**	11.3	48.7	39.6	63.1 **	19.9 ^§^	NA	12.7	35
**Median DOR, months**	NR	NR	NR	11.1 **	23.1 ^§^	NA	NR	NA
**PFS**	24-month: 30%	48-month: 33%	6-month: 63.5%	60-month: 31.8%	24-month: 40.6% ^§^	2-year: 64%	1-year: 74%	2-year: 54%
**OS**	36-month: 81%	48-month: 45.3%	6-month: 86.3%	60-month: 42.6%	24-month: 50.5% ^§^	2-year: 78%	1-year: 86%	2-year: 75%

ASCT: autologous stem cell transplant, RT: radiotherapy, ORR: overall response rate, CR: complete remission, DOR: duration of response, PFS: progression-free survival, OS: overall survival, NA: not available, NR: not reached. * PMLBCL and transformed follicular lymphoma. ** All patients. ^§^ All patients.
